# Retrospective examination of injuries and physical fitness during Federal Bureau of Investigation new agent training

**DOI:** 10.1186/1745-6673-6-26

**Published:** 2011-10-09

**Authors:** Joseph J Knapik, Anita Spiess, David Swedler, Tyson Grier, Keith Hauret, James Yoder, Bruce H Jones

**Affiliations:** 1 U.S. Army Institute of Public Health, Aberdeen Proving Ground, Maryland, USA; 2 Johns Hopkins University, Bloomberg School of Public Health, Baltimore, MD, USA; 3 Federal Bureau of Investigation, Human Resources Division, Office of Medical Services, Health Care Programs Unit, Washington, DC, USA

**Keywords:** Overuse, trauma, law enforcement, physical training, gender, 1.5-mile run

## Abstract

**Background:**

A retrospective examination was conducted of injuries, physical fitness, and their association among Federal Bureau of Investigation (FBI) new agent trainees.

**Methods:**

Injuries and activities associated with injuries were obtained from a review of medical records in the medical clinic that served the new agents. A physical fitness test (PFT) was administered at Weeks 1, 7 and 14 of the 17-week new agent training course. The PFT consisted of push-ups, sit-ups, pull-ups, a 300-meter sprint, and a 1.5-mile run. Injury data were available from 2000 to 2008 and fitness data were available from 2004 to early 2009.

**Results:**

During the survey period, 37% of men and 44% of women experienced one or more injuries during the new agent training course (risk ratio (women/men) = 1.18, 95% confidence interval = 1.07-1.31). The most common injury diagnoses were musculoskeletal pain (not otherwise specified) (27%), strains (11%), sprains (10%), contusions (9%), and abrasions/lacerations (9%). Activities associated with injury included defensive tactics training (48%), physical fitness training (26%), physical fitness testing (6%), and firearms training (6%). Over a 6-year period, there was little difference in performance of push-ups, sit-ups, pull-ups, or the 300-meter sprint; 1.5-mile run performance was higher in recent years. Among both men and women, higher injury incidence was associated with lower performance on any of the physical fitness measures.

**Conclusion:**

This investigation documented injury diagnoses, activities associated with injury, and changes in physical fitness, and demonstrated that higher levels of physical fitness were associated with lower injury risk.

## Background

The Federal Bureau of Investigation (FBI) is tasked with upholding and enforcing the criminal laws of the United States, protecting the United States against terrorist and foreign intelligence threats, and providing law enforcement and investigative leadership and services to federal, state, municipal, and international agencies and partners [1]. To accomplish these and other tasks, the FBI Academy at Quantico, Virginia, trained an average of 700 new agents each year from 2000 to 2008. During this period, the new agent course involved about 850 hours of instruction covering academics (such as fundamentals of law, behavioral science, investigative and intelligence techniques, interviewing, and forensic science), case scenarios, firearms, operational skills, and other activities.

As in athletics and the military, physical training is an important part of the FBI new agent training program. New agents undergo about 90 hours of defensive tactics training in which agents learn procedures for defending themselves against physical threats and apprehending suspects. New agents are expected to maintain a level of physical fitness that allows them to accomplish the physical tasks they are expected to perform. They are required to pass a physical fitness test and to perform regular exercise training, either individualized or in groups.

Primarily because of the physical tasks they perform, new agents will face some risk of injury during their training program. In April 2008, the FBI Health Programs Unit requested the assistance of the US Army Institute of Public Health (USAIPH) in investigating injuries at the FBI Academy. The initial concern was a recent outbreak of exertional rhabdomyolysis, but discussions between the FBI and USAIPH resulted in a broader goal, which was to examine all injuries and how physical fitness might be associated with these injuries; no previous effort had been made to systematically examine these issues in FBI new agent training. Thus, the purpose of the investigation described here was to report injuries, physical fitness, and the association of injuries and physical fitness in FBI new agent trainees.

## Methods

This project involved a retrospective examination of injuries and physical fitness among students in FBI new agent training at the FBI Academy in Quantico, Virginia. Descriptive information was obtained from available databases and the association between injuries and physical fitness was examined. The project was reviewed and approved as a public health practice project [2] by the Human Use Review Committee of the FBI, Washington DC.

### Injury Data

All FBI new agents received an initial medical examination to determine fitness for duty prior to arrival at the FBI Academy. This included an evaluation of injuries that might affect their performance during training. While at the FBI Academy, medical care was provided at the FBI Health Clinic. Medical care providers at the clinic routinely entered information on new agent medical encounters into a database. Medical encounters from 1 October 1999 to 30 September 2008 were examined in this database by trained and experienced personnel who determined if the encounter was for an injury (defined below) or for other medical care. For each injury encounter, extracted information included the date of visit, type of visit (new injury visit or follow-up on a previous visit), diagnosis, anatomical location, and activity associated with the injury. The number of new agents training at the FBI Academy in the injury survey period was obtained from the FBI Human Resources Division. Only the total number of new agents was available and there was no breakdown by gender.

An injury case was a new agent who sustained physical damage to the body and sought medical care one or more times during the survey period. Injuries were grouped by "type," which was determined from descriptive information in the medical notes and by the specific diagnosis. Injury types included 1) overuse injury, 2) traumatic injury, 3) any injury, 4) environmental injury and 5) rhabdomyolysis. Overuse injuries were presumably related to long-term repetitive energy exchanges, resulting in cumulative microtrauma. Specific overuse diagnoses included stress fractures, tendonitis, bursitis, fasciitis, muscle injury presumably due to overuse (strain), joint injury presumably due to overuse (sprain), retropatellar pain syndrome, impingement, degenerative joint conditions, shin splints, and musculoskeletal pain (not otherwise specified but with pain developing over time). A traumatic injury was presumably due to sudden energy exchanges (acute event), resulting in abrupt overload with tissue damage. Specific traumatic diagnoses included muscle injury due to acute event (strain), joint injury due to an acute event (sprain), dislocation, fracture, blister, abrasion, laceration, contusions, closed head injury/concussion, and pain (not otherwise specified, but due to an acute event). An environmental injury was presumably due to exposure to weather, animals, or chemicals, resulting in physical damage to the body. Environmental and other injury diagnoses included heat-related injuries, animal bites, chemical exposures and others. Any injury combined the overuse and trauma diagnoses as described above, but excluded environmental/other injuries. The "any injury" type included primarily musculoskeletal injuries, but also included dermatological insults (e.g., blisters, abrasions, lacerations). Because of a special interest in rhabdomyolysis at the FBI academy, rhabomyolysis occurrences were categorized separately. To be classified as rhabdomyolysis, the medical record had to have included the diagnosis of "rhabdomyolysis" or "possible rhabdomyolysis," and/or reported a creatine kinase level exceeding 1,000 U/L.

New injuries were first medical encounters with a new agent that resulted in a particular injury diagnosis at a particular anatomical location. Follow-ups were subsequent medical encounters for the same injury at the same anatomical location as the new injury (first encounter). If follow-ups occurred, they were used in conjunction with the initial encounter to determine the final diagnosis for a specific injury. Thus, an initial diagnosis could be changed as a result of a more specific diagnosis at a higher level of medical care.

### Physical Fitness Data

Physical fitness test (PFT) data were obtained from an existing database in the Physical Training Unit of the FBI Academy. Every new agent entering the FBI Academy was required to take and pass the PFT as a graduation requirement. PFTs were administered within 2 days of arrival at the Academy (Week 1), at Week 7, and at Week 14 of the new agent training course. If a new agent passed the Week 1 or Week 7 test, they were not required to take the Week 14 test. PFT data from 31 May 2004 through 1 March 2009 were obtained. The database contained scores on 5 events, total points on the test, and the gender of the new agent. The PFT consisted of 4 "scored" events: push-ups to exhaustion (continuous motion), 1-minute bent-leg sit-ups, 300-meter sprint, and 1.5-mile run. At least 5 minutes of rest were provided between events. Points were assigned to various levels of performance on each PFT event (some performance levels result in negative point, i.e., points < 0). To pass the PFT, a point score of 12 was required with at least 1 point on each event. Pull-ups to exhaustion were also tested, but this event was not included in the FBI's standard calculation of the total point score. Details on the PFT and the scoring system can be found on-line [3].

### Data Analysis

Data were compiled and analyzed using the Statistical Package for the Social Sciences (SPSS), version 16.0.1. Data from the medical records and the PFT database were combined and a deidentifed database was created. Data from the PFT database provided gender-specific denominators for calculating injury rates, and thus injury incidence could be calculated from fiscal year (FY) 2004 to FY 2008 (a FY is from October 1^st ^one year to September 30^th ^the next year). For all injury types, injury incidences were calculated as new agent trainees with ≥ 1 injuries divided by total number of new agents, described as a percentage. Chi-square statistics were used to compare injury incidences between men and women.

Scores on the Week 1 and Week 7 PFT were compared using a paired t-test. Average scores for PFT events were also plotted by FY and linear regression modeling was used to examine the changes in each event over the years. The slope of the linear regression equation provided the least squares estimate of the changes in the average event scores over the years. The r^2 ^provided an estimate of the goodness of fit of the regression equation.

Chi-square statistics, linear trend tests, and logistic regression were used to examine the associations between the physical fitness measures and injury incidence. The dependent variable in the logistic regressions was the presence or absence of any injury. All fitness events were entered into the logistic regression models as quartiles (four approximately equal-sized groups). Simple contrasts with a baseline variable (defined with an odds ratio of 1.00) were used to describe changes in injury risk across strata.

## Results

There were a total of 6,298 new agents who were involved in new agent training during the injury survey period. These new agents experienced a total of 4,616 new injuries with 1,026 follow-ups. Table 1 shows the number of new injury cases and follow-ups by diagnosis. It was not possible to separate the injuries by gender because the medical records did not contain these data. Overuse injuries made up 15% of new injury cases, traumatic injuries 70%, and environmental/other injuries 15%. The diagnosis with largest number of cases was musculoskeletal pain associated with trauma. These cases involved encounters where an individual reported pain in a specific musculoskeletal location from a traumatic event, but no specific diagnosis was found in the medical record. Next in rank order of the number of new cases were traumatic injuries to muscles (strains), traumatic injuries to joints (sprains), contusions, and abrasions/lacerations. With regard to anatomical locations, the head accounted for 16% of new injury cases, the upper body 42%, and the lower body 37%. The most common anatomical sites of new injury cases were the knees (9.9%), shoulders (8.2%), thigh (7.9%), face (6.7%), ankle (5.7%), chest (5.6%), fingers (5.3%), low back (4.7%), foot (4.3%), neck (3.8%), shin (3.6%), elbow (3.1%), hand (3.1%), calf (2.7%), wrist (2.6%) and head (2.0%).

**Table 1:** Injury Cases by Type and Diagnoses

Type	Diagnosis	New Injuries		Follow-Ups	
		Cases (n)	Proportion of Total Cases (%)	Cases (n)	Proportion of Total Cases (%)
Overuse	Stress fracture	2	0.0	2	0.2
	Tendonitis	79	1.7	36	3.5
	Degenerative joint disease	1	0.0	0	0.0
	Bursitis	10	0.2	14	1.4
	Fasciitis	12	0.3	7	0.7
	Retropatellar pain syndrome	22	0.5	11	1.1
	Impingement	21	0.5	13	1.3
	Muscle injury (overuse strain)	66	1.4	24	2.3
	Joint injury (overuse sprain)	18	0.4	5	0.5
	Musculoskeletal pain (overuse)	340	7.4	95	9.3
	Shin splints	122	2.6	48	4.7
Traumatic	Muscle injury (traumatic strain)	520	11.3	175	17.1
	Joint injury (traumatic sprain)	437	9.5	146	14.2
	Musculoskeletal pain (traumatic)	1250	27.1	159	15.5
	Other traumatic injury	20	0.4	4	0.4
	Dislocation	46	1.0	24	2.3
	Bone Fracture	32	0.7	28	2.7
	Tooth Fracture	35	0.8	0	0.0
	Nasal Fracture	9	0.2	6	0.6
	Blister	30	0.6	2	0.2
	Abrasion or laceration	404	8.8	39	3.8
	Contusion	428	9.3	78	7.6
	Neurological	16	0.3	3	0.3
	Closed Head Injury/Concussion	19	0.4	15	1.5
Environmental or Other	General heat-related injury	25	0.5	7	0.7
	Heat exhaustion	30	0.6	5	0.5
	Heat stroke	3	0.1	0	0.0
	Rhabdomyolysis	14	0.3	14	1.4
	Exertion-related	102	2.2	5	0.5
	Dehydration	34	0.7	6	0.6
	Insect bites or stings	202	4.4	21	2.0
	Other animal bite	2	0.0	0	0.0
	Other, environ/toxic injury	2	0.0	1	0.1
	Chemical Burn (Capsicum Spray)	244	5.3	27	2.6
	Thermal Burn (Shell Casing)	17	0.4	3	0.3
	Cold Injury	1	0.0	0	0.0
Unknown		1	0.0	3	0.3

Table 2 shows the number of injury cases by the training activity associated with the injury. Almost half the new injury cases were associated with defensive tactics training and another quarter of the cases were associated with physical fitness training. Together, defensive tactics and physical fitness training accounted for 74% of activities associated with injury.

**Table 2:** Injury Cases by Training Activity

Activity	New Injuries		Follow-Ups	
	Cases (n)	Proportion of Total Cases (%)	Cases (n)	Proportion of Total Cases (%)
Defensive Tactics Training	2206	47.8	442	43.1
Physical Fitness Training	1218	26.4	351	34.2
Physical Fitness Testing	262	5.7	63	6.1
Firearms Training	253	5.5	34	3.3
Off-Duty, at Academy	54	1.2	10	1.0
Off Duty, Not at Academy	43	0.9	2	0.2
Operational Skills Training	79	1.7	26	2.5
Sports	31	0.7	3	0.3
Other	54	1.2	3	0.3
Unknown	416	9.0	92	9.0

Table 3 shows injury incidence by gender and type. Complete gender-specific denominators were available only for FY 2004 through early 2009 (from the PFT). Only data from FY2004 to FY2008 were included in Table 3 so that 5 complete years of data could be included. Compared to men, women had a significantly higher incidence of any injury, overuse injury, traumatic injury, environmental injury, and rhabdomyolysis. Gender differences were much smaller for any injury and traumatic injury than for the other injury types.

**Table 3:** Injury Incidence (FY 2004-FY 2008) by Injury Type and Gender

Injury Type	Men (% injured) n = 2,555	Women (% injured) n = 693	Risk Ratio-Women/Men (95%CI)	**p-value**^**a**^
Any Injury	37.1	44.0	1.19 (1.08-1.31)	< 0.01
Overuse Injury	7.8	12.4	1.58 (1.25-2.01)	< 0.01
Traumatic Injury	33.3	38.7	1.16 (1.04-1.30)	< 0.01
Environmental Injury	3.2	5.2	1.62 (1.10-2.38)	< 0.01
Rhabdomyolysis	0.2	1.0	5.15 (1.64-16.1)	< 0.01

^a^From chi-square statistic

Table 4 compares the results of the Week 1 and Week 7 PFTs. Week 1 PFT scores were available for 2,837 men and 771 women, but Table 4 contains only those who took both tests. Only 398 men and 81 women took the Week 14 test, so these data are not shown. There were significant improvements on all test events from Week 1 to Week 7. Women improved more than men on both a relative (%) and an absolute basis for all events excluding pull-ups.

**Table 4:** Entry-Level Physical Fitness and Changes in Physical Fitness from Week 1 to Week 7

Gender	Event	N	Week 1 Mean ± SD	Week 7 Mean ± SD	Δ	**%Δ**^**a**^	**p-value**^**b**^
Men	Push-Ups (n)	2,576	37.7 ± 9.4	40.6 ± 8.9	2.9	7.7	< 0.01
	Sit-Ups (n)	2,580	45.9 ± 5.4	50.2 ± 4.2	4.3	9.4	< 0.01
	300-Meter Sprint (sec)	2,578	46.3 ± 2.6	45.3 ± 2.4	-1.0	2.2	< 0.01
	1.5-Mile Run (min)	2,551	11.3 ± 1.0	10.8 ± 0.9	-0.5	4.4	< 0.01
	Total Score (points)	2,527	14.6 ± 5.2	18.8 ± 4.9	4.2	28.8	< 0.01
	Pull-Ups (n)	2,546	7.7 ± 4.4	8.4 ± 4.5	0.7	9.1	< 0.01
Women	Push-Ups (n)	655	19.8 ± 8.9	23.6 ± 7.3	3.8	19.2	< 0.01
	Sit-Ups (n)	655	44.5 ± 6.9	49.8 ± 4.2	5.3	11.9	< 0.01
	300-Meter Sprint (sec)	654	56.6 ± 3.7	54.9 ± 3.5	-1.7	3.0	< 0.01
	1.5-Mile Run (min)	649	12.7 ± 1.2	12.0 ± 0.9	-0.7	5.5	< 0.01
	Total Score (points)	645	14.1 ± 5.8	19.5 ± 5.6	5.4	38.3	< 0.01
	Pull-Ups (n)	641	0.9 ± 1.9	1.1 ± 2.2	0.2	22.2	< 0.01

^a^Calculated as (Week 7-Week 1)/Week 1 × 100%

^b^From paired t-test

**Figure 1:**
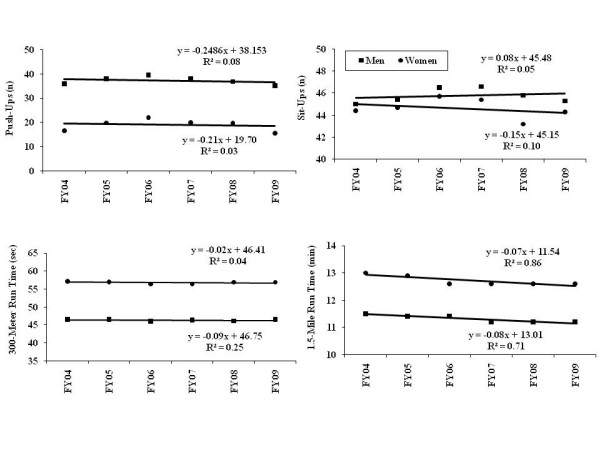
**Physical Fitness Test Scores by Fiscal Year (FY) from 2004 to 2009**. Push-Ups Upper Left, Sit-Ups Upper Right, 300-Meter Run Lower Left, 1.5 Mile Run Lower Right. Linear regression equations and squared correlation coefficient are shown for each event.

Figure 1 graphically displays the Week 1 scores for each PFT event by FY. Figure 1 also shows the linear regression equations and the r^2 ^values for each PFT event. Push-ups, sit-ups and 300-meter sprint scores showed little change from 2004 to 2009. On the other hand, the average 1.5-mile run time became progressively faster over the period. Based on the linear regression slope, run times were about 4 seconds per year faster or about 24 seconds faster in FY 2009 compared to FY 2004.

Table 5 shows associations between entry level fitness (Week 1 PFT) and any injury. Only new agents who had graduated at the time of the injury data collection were included so that the entire training period could be included (equating time at risk). Pull-ups were not examined for the women because only 29% of women had ≥ 1 pull-up recorded. Among men, higher injury incidence was associated with lower performance on push-ups, sit-ups, the 300-meter sprint, the 1.5-mile run, the total score, and pull-ups. Among women, higher injury incidence was associated with lower performance on the 1.5-mile run and the total score; weaker associations were shown between injuries and push-up, sit-up, and 300-meter sprint performance, but nonetheless lower performance on these events was still associated with higher injury risk.

**Table 5:** Associations between Physical Fitness and the Incidence of Any Injury

Gender	Fitness Variable	Strata	N	Injured (%)	p-values from chi-square/trend tests	Odds Ratios (95%CI) from Logistic Regression
Men	Push-Ups	1-31 repetitions	522	46.6		1.39 (1.10-1.77)
		32-37 repetitions	571	37.8	0.01/	0.97 (0.77-1.23)
		38-43 repetitions	590	39.2	0.02	1.03 (0.81-1.30)
		44-75 repetitions	595	38.5		1.00
	Sit-Ups	0-42 repetition	534	46.8		1.58 (1.23-2.02)
		43-46 repetitions	662	35.5	< 0.01/	0.99 (0.77-1.25)
		47-49 repetitions	560	44.3	0.02	1.43 (1.12-1.82)
		50-62 repetitions	525	35.8		1.00
	300-Meter Sprint	38.2-44.5 seconds	577	36.0		1.00
		44.6-46.2 seconds	561	40.5	0.02/	1.21 (0.95-1.53)
		46.3-47.9 seconds	555	39.6	< 0.01	1.17 (0.92-1.48)
		48.0-56.6 seconds	588	45.2		1.47 (1.16-1.85)
	1.5-Mile Run	7.63-10.70 minutes	569	27.6		1.00
		10.71-11.30 minutes	551	31.5	< 0.01/	1.18 (0.93-1.51)
		11.31-11.90 minutes	561	31.5	< 0.01	1.21 (0.95-1.55)
		11.91-20.00 minutes	576	42.3		2.01 (1.58-2.54)
	Total Physical	-4-10 points	522	46.6		1.89 (1.45-2.41)
	Fitness Test Score^a^	11-14 points	571	37.8	0.01/	1.07 (0.85-1.33)
		15-17 points	590	39.2	0.01	1.07 (0.84-1.36)
		18-36 points	595	38.5		1.00
	Pull-Ups	0-4 repetitions	592	42.9		1.43 (1.10-1.86)
		5-7 repetitions	563	42.1	0.04/	1.38 (1.06-1.80)
		8-11 repetitions	699	39.6	< 0.01	1.25 (0.98-1.61)
		12-27 repetitions	406	34.5		1.00
						
Women	Push-Ups	0-13 repetitions	142	52.1		1.51 (0.96-2.35)
		14-18 repetitions	128	51.6	0.17/	1.47 (0.93-2.33)
		19-24 repetitions	178	43.8	0.03	1.08 (0.71-1.65)
		25-55 repetitions	174	42.0		1.00
	Sit-Ups	2-41 repetition	152	52.6		1.45 (0.94-2.24)
		42-45 repetitions	162	51.9	< 0.01/	1.41 (0.92-2.16)
		46-48 repetitions	128	38.3	0.05	0.81 (0.51-1.29)
		49-59 repetitions	180	43.3		1.00
	300-Meter Sprint	45.6-54.4 seconds	161	44.7		1.00
		54.5-56.6 seconds	150	46.7	0.19/	1.08 (0.69-1.69)
		56.7-59.2 seconds	157	42.0	0.20	0.90 (0.57-1.40)
		59.3-72.5 seconds	154	53.9		1.45 (0.93-2.25)
	1.5-Mile Run	9.02-11.92 minutes	159	46.5		1.00
		11.93-12.63 minutes	140	45.0	0.02/	0.94 (0.60-1.48)
		12.64-13.42 minutes	162	39.5	0.19	0.75 (0.48-1.17)
		13.43-20.00 minutes	158	56.3		1.53 (1.00-2.24)
	Total Physical Fitness Test Score^a^	-2-10 points	167	54.5		1.81 (1.17-2.80)
		11-14 points	177	48.6	0.04/	1.43 (0.93-2.19)
		15-17 points	118	42.4	< 0.01	1.11 (0.69-1.79)
		18-36 points	163	39.9		1.00

^a^Negative points are possible in the scoring system

## Discussion

The investigation reported here identified common injury diagnoses, activities associated with injury, comparisons of injury incidence rates in men and women, and the associations between fitness and injuries in FBI new agent training. Thirty-seven percent of men and 44% of women experienced one or more injuries in training. Defensive tactics and physical fitness training were associated with 74% of all injuries. A 6-year examination of temporal trends in physical fitness showed little change in push-up, sit-up, or 300-meter sprint performances, but the average 1.5-mile run performance did improve. Lower levels of physical fitness were associated with higher injury risk.

### Injuries

Besides musculoskeletal pain, the largest numbers of injuries were from strains, sprains, contusions, and abrasions/lacerations. These are common injuries in physically active groups of individuals who are involved in running, sports, recreational activities, and military training [4-14]. Only a few cases of more serious traumatic injuries such as bone fractures, dislocations, and subluxations occurred; these totaled less than 2% of all injuries. In runners and collegiate athletes, fractures, subluxations, dislocations have accounted for 3% to 13% of all injuries [4,6,7,9-12]. Less serious injuries like abrasions/lacerations and contusions each accounted for about 9% of new agent injuries, which is comparable to that reported in the literature: 8% to 11% for abrasions/lacerations [7,10,12] and 6% to 24% for contusions [6,9,11,12]. This suggests that compared to other groups of active individuals, new agent training has a lower proportion of more serious injuries. However, it is possible that had the musculoskeletal pain (not otherwise specified) cases been diagnosed at higher levels of medical care, these may have been added to other diagnostic categories.

Among new agent trainees only 15% of injuries were classified as overuse with 70% classified as traumatic. With regard to specific injuries, tendonitis accounted for less than 2% of FBI new agent injuries, but in runners, college athletes, and military trainees this injury accounts for 5% to 12% of all injuries [4-6,8,10,13]. In military basic combat training (BCT) overuse-type injuries account for about 75% of all injuries [15]. Contrasting the activity patterns in military training with that of FBI new agent training might assist in accounting for some of these differences. In BCT, recruits perform virtually all physical training as a group, regardless of fitness level. During running activities individuals of similar fitness run together [16] but with this single exception: all other physical and operational training is conducted together, regardless of fitness level. The basic rationale is to keep the recruits together to build fitness and operational competence while at the same time developing morale (esprit de corps) and teamwork, all under the guidance of a knowledgeable leader, the drill sergeant. In contrast, most FBI new agent trainees performed physical fitness training on their own. A proportion of new agents who failed the first PFT were required to attend a group physical training program three times per week. It is likely that the individualized physical training performed by most FBI new agent resulted in fewer overuse injuries since the intensity, frequency, and duration was determined by the individual. In contrast to physical fitness training, defensive tactics instruction was conducted in a group, with all new agents training together. Defensive tactics involved boxing, self-defense techniques, and subduing suspects (wrestling, grappling, handcuffing). The violent nature of these activities likely led to a higher incidence of traumatic injuries. Another consideration with regard to distinguishing between overuse and traumatic injuries might be workman's compensation that all FBI new agents are eligible for. There is some incentive to link specific injuries to specific causes (rather than note that the injury had occurred gradually over time) because workman's compensation forms require a "cause" of injury. Thus, it may be difficult to truly separate overuse and traumatic injuries in this population.

In the present investigation, 37% of cases involved the lower body and 52% involved the upper body. In sports and recreational activities, the lower body is the site of over 50% and up to 84% of all injuries [7,11,17,18]. In military basic training, 77% to 88% of injuries are to the lower body [13,15], and in military infantry operational training about 50% to 60% of injuries involve the lower body [19,20]. Much of military training involves the lower body in activities like running for physical training and patrols on foot while carrying equipment (road marching), walks to training area, drill and ceremony, and the like. In contrast, many injuries in FBI new agent training are associated with defensive tactics which involved largely the upper body.

From an injury-prevention standpoint, the most important information in the medical records is how the injury occurred (i.e., activity or cause). Since almost 50% of injuries were associated with defensive tactics, this is the obvious focus for injury prevention efforts. Given the emphasis in the curriculum and the physical involvement, it is reasonable that most of the injuries would occur in defensive tactics and physical training. We observed that many safety features were in place at the FBI academy during defensive tactics training. Examples are in boxing, the use of boxing gloves, headgear, and mouthguards. During other defensive tactics training, agents practiced on cushioned mats, which offered some protection during falls and takedowns. Nonetheless, additional efforts to reduce injuries in defensive tactics should be explored.

### Physical Fitness

Over the 6-year period from FY 2004 to FY 2009 there was virtually no change in the performance of push-ups, sit-ups, or the 300-meter sprint, but performance on the 1.5-mile run did improve. This is in contrast to studies of US Army Basic Combat Training and international data on youth that show declines in running performance [21-23]. Since 2005, statistical reviews of physical fitness testing have been provided periodically to FBI field offices [24]. These reviews noted the number and percent of new agent trainees who have failed the initial PFT and compares failures among field offices. Field offices are charged with assuring that new agent trainees sent to the FBI Academy are physically prepared, including prepared to pass the PFT. It is possible that these reports have called more attention to fitness issues at these field offices, helping maintain pre-academy push-up, sit-up and 300-meter sprint performance and improving 1.5-mile run times.

### Associations between Fitness and Injuries

Because of the limitations in defining and separating overuse and traumatic injuries mentioned above, the any injury variable was used to examine the association between fitness and injuries. The data generally showed that higher levels of physical fitness were associated with lower levels of injury. This agrees well with investigations in military basic training [13,15,25-29] and studies of infantry soldiers [19,20,30]. However, the FBI new agent data do not agree with most studies of free living individuals [17,31-36], which generally find that individuals with higher fitness levels have higher injury incidence.

One of the common characteristics of military basic training and FBI new agent training is that individuals perform many physical activities with their fellow trainees. In the present investigation, about 50% of all activities were associated with defensive tactics which all new agent trainees perform together and thus are all exposed to similar risks. About 25% of injuries were associated with new agent physical training. New agent trainees who fail the initial PFT are required to attend supervised physical training three times per week and these new agent trainees would be performing very similar training. New agent trainees who pass the initial PFT are allowed to perform physical training on their own. Nonetheless, the types of physical training performed by these new agent trainees was likely similar to that of other new agent trainees and this training likely involved both strength and aerobic training and focused on passing the PFT in Week 7. It is possible that the relationship between low fitness and higher injury risk can be demonstrated in military basic training and in new agent training (but not in civilian groups) because in basic training and new agent training, the level and type of physical training are similar among participants.

Law enforcement agencies in the United States and abroad can benefit from the results of this study by emphasizing physical fitness in the training of new officers. This study and previous ones [13,15,25-27,29] have shown that individuals who arrive for training at a higher level of physical fitness are less likely to be injured. Thus, individuals should be strongly encouraged to attain a high level of fitness prior to entry into law enforcement training. Consideration should also be given to developing pre-law enforcement training physical training programs, especially for individuals who display low initial levels of physical fitness. Previous military studies have shown that such structured fitness-orientated programs result in lower injury rates once individuals begin boarder military training [37,38].

### Limitations

Injury diagnoses were limited to descriptions in the medical records. Many of these did not involve diagnostic tests which would have provided more definitive diagnoses. The largest category of injury was traumatic musculoskeletal pain, which was not more specifically defined. This category involved encounters where an individual reported pain in a specific musculoskeletal location but no more specific diagnosis was found in the medical record. Thus, the actual incidence of specific diagnoses was very likely underestimated. Nonetheless, the data provides a comprehensive look at the available medical visits and shows a high incidence of strains, sprains, contusions and lacerations which are common injuries in physically active populations [4-14].

Injury risk was probably slightly underestimated. The analysis assumes that all agents completed the 17-week training course, but some new agent trainees did not for various reasons. Agents who dropped out of training had less time at risk (i.e., less time exposed to the hazards of training). To obtain an idea of the size of this error, we obtained group data (individual data were not available) on the number of new agent trainees who did not complete the course in FY 2005 through FY 2007. There was only a 2.9% to 3.8% drop out rate. The effect of drop outs on the data is likely small.

## List of abbreviations

FBI: Federal Bureau of Investigation; USAIPH: United States Army Institute of Public Health; PFT: Physical Fitness Test; FY: Fiscal year.

## Competing interests

The authors declare that they have no competing interests.

## Authors' contributions

JJK was involved in the conception and design, data collection, data analysis, data interpretation, manuscript writing, and final approval of manuscript. AS was involved in the data collection, data interpretation, and final approval of manuscript. DS contributed to the data collection, data interpretation, and final approval of manuscript. TG was instrumental in the data collection, data interpretation, and final approval of manuscript. KGH was involved in the conception and design of the project, data collection, data interpretation, manuscript writing, and final approval of manuscript. JY was involved in the conception and design, data interpretation, and final approval of manuscript. BHJ contributed to the conception and design, data interpretation, final approval of manuscript
